# Green and Facile Synthesis of Metal-Organic Framework Cu-BTC-Supported Sn (II)-Substituted Keggin Heteropoly Composites as an Esterification Nanocatalyst for Biodiesel Production

**DOI:** 10.3389/fchem.2020.00129

**Published:** 2020-03-18

**Authors:** Qiuyun Zhang, Dan Ling, Dandan Lei, Jialu Wang, Xiaofang Liu, Yutao Zhang, Peihua Ma

**Affiliations:** ^1^School of Chemistry and Chemical Engineering, Anshun University, Anshun, China; ^2^Engineering Technology Center of Control and Remediation of Soil Contamination of Provincial Science & Technology Bureau, Anshun University, Anshun, China; ^3^School of Resource and Environmental Engineering, Anshun University, Anshun, China; ^4^Food and Pharmaceutical Engineering Institute, Guiyang University, Guiyang, China; ^5^School of Chemistry and Chemical Engineering, Guizhou University, Guiyang, China

**Keywords:** heteropolys, Cu-BTC, nanocomposites, esterification, biodiesel

## Abstract

In the present study, metal-organic framework Cu-BTC-supported Sn (II)-substituted Keggin heteropoly nanocomposite (Sn_1.5_PW/Cu-BTC) was successfully prepared by a simple impregnation method and applied as a novel nanocatalyst for producing biodiesel from oleic acid (OA) through esterification. The nanocatalyst was characterized by Fourier transform infrared spectrometry (FTIR), wide-angle X-ray diffraction (XRD), scanning electron microscopy (SEM), transmission electron microscopy (TEM), nitrogen adsorption-desorption, thermogravimetrics (TG), and NH_3_-temperature-programmed desorption (NH_3_-TPD). Accordingly, the synthesized nanocatalyst with a Sn_1.5_PW/Cu-BTC weight ratio of 1 exhibited a relatively large specific surface area, appropriate pore size, and high acidity. Moreover, an OA conversion of 87.7% was achieved under optimum reaction conditions. The nanocatalyst was reused seven times, and the OA conversion remained at more than 80% after three uses. Kinetic study showed that the esterification reaction followed first-order kinetics, and the activation energy (*E*_*a*_) was calculated to be 38.3 kJ/mol.

## Introduction

Nowadays, fossil fuel resource demand is expanding progressively due to industrial growth and constant population rise. Meanwhile, the utilization of fossil fuels along with environmental pollution and global warming has led to the consideration of alternative energy sources like biofuels (Bhanja and Bhaumik, 2016; Long et al., 2019; Negm et al., 2019; Xu et al., 2019; Li et al., 2020). Among the various biofuels, biodiesel is considered to be the most promising renewable fuel, possibly due to its biodegradable, non-toxic, and environmentally friendly features (Al-Saadi et al., 2018; Li H. et al., 2019). Further, the typical way of producing biodiesel is the catalytic esterification of free fatty acids or transesterification of triglyceride with an alcohol in the presence of a homogeneous/heterogeneous catalyst (Mahmoud, 2019). A homogeneous acid catalyst, such as HCl, H_2_SO_4_, or H_3_PO_4_, can catalyze esterification with high activity; however, the major drawbacks of homogeneous acid catalysts are the generation of a huge amount of chemical wastewater and the high cost for catalyst separation and non-reusability (Zhang et al., 2019a). Therefore, heterogeneous solid catalysts have been widely applied to catalyze the esterification reaction for biodiesel synthesis.

One option is to use hetropolyacids as strong Brønsted acid catalysts for catalytic esterification and transesterification reactions to produce biodiesel with high conversions (Talebian-Kiakalaieh et al., 2013; Sun et al., 2015; Xie and Wan, 2019a). Unfortunately, hetropolyacids have certain disadvantages, such as good solubility in polar media and a low surface area (Parida and Mallick, 2007; Ekinci and Oktar, 2019). Therefore, supporting hetropolyacids on porous supports is an interesting approach to produce heterogeneous catalysts, since it can provide high surface area and insolubility in the polar solvent.

Various types of supported hetropolyacid catalysts have been utilized. Montmorillonite K10 (Nandiwale and Bokade, 2014), Nb_2_O_5_ (da Conceiçao et al., 2017), and carbon (Ghubayra et al., 2019) can be used as supports, but they were more or less subject to the disadvantages of weak interaction between object and host, low stability, high-cost synthesis, etc. By contrast, metal-organic frameworks (MOFs) provide excellent support, having the features of stability, adjustable tunnels, ultra-high specific surface area, and high catalytic efficiency (Kang et al., 2019; Li D. D. et al., 2019). Examples are PTA@MIL-53 (Fe) (Nikseresht et al., 2017), AILs/HPW/UiO-66-2COOH (Xie and Wan, 2019b), and ZnFe_2_O_4_/MIL-100(Fe) (Hu et al., 2019). Meanwhile, our previous studies showed the esterification of oleic acid or lauric acid with methanol over silicotungstic acid and nickel salts of Keggin-type heteropolyacids encapsulated into metal-organic framework (UiO-66) hybrid nanocatalysts that had excellent activity and reusability (Zhang et al., 2019b, 2020).

To date, there have been no reports on carrying out esterification reaction for biodiesel production using metal-organic framework Cu-BTC-supported Sn (II)-substituted Keggin heteropolyacids as nanocatalysts. Thus, in this work, we successfully synthesized a series of nanocatalysts consisting of Sn (II)-substituted 12-tungstophosphoric acid on a Cu-BTC matrix (Sn_1.5_PW/Cu-BTC-x) at different ratios and used those nanocatalysts for producing biodiesel from OA with methanol. The characterization of synthesized nanocomposites was done using FTIR, XRD, SEM, TEM, nitrogen adsorption-desorption, TG, and NH_3_-TPD. Further, the effect of different reaction parameters such as the molar ratio of methanol to OA, amount of catalyst, and reaction time and temperature were investigated to optimize the esterification conditions. Kinetic studies of the OA esterification reaction over the Sn_1.5_PW/Cu-BTC nanocatalyst were studied. Finally, the reusability of those composites was also studied for seven successive runs.

## Experimental Section

### Materials and Synthesis

All chemicals were obtained from commercial sources and used without further purification. Copper (II) acetate monohydrate (Cu(CO_2_CH_3_)_2_·H_2_O, AR), 1,3,5-benzenetricarboxylic acid (H_3_-BTC) (AR), tin chloride dehydrate (SnCl_2_·2H_2_O, AR), and 12-tungstophosphoric acid (H_3_PW_12_O_40_, HPW, AR) were purchased from Shanghai Aladdin Industrial Inc. Oleic acid (OA, AR), N,N-dimethylformamide (DMF, AR), acetic acid (AR), absolute ethanol (AR), and anhydrous methanol (AR) were purchased from Sinopharm Chemical Reagent Co., Ltd.

Firstly, Sn_1.5_PW_12_O_40_ (Sn_1.5_PW) salts were prepared by stirring an aqueous solution containing the HPW and SnCl_2_ at room temperature for 3 h; then, the obtained mixture was dried overnight at 120°C, according to our previous reports (Zhang et al., 2019a). Second, Cu-BTC was prepared from 0.06 g of copper (II) acetate monohydrate and 0.6 g of acetic acid dissolved in 6 mL distilled water, and 0.16 g of H_3_-BTC dissolved in 6 mL of DMF was added dropwise from above the mixture solution. The resulting solution continued to be stirred for 3 h at room temperature. Then, the precipitate was collected by centrifugation and washed with 50 mL of hot ethanol two times and hot water once, and the blue powder was dried at 120°C for 12 h, according to the literature (Na et al., 2012). Finally, Cu-BTC-supported Sn (II)-substitute phosphotungstic acid catalysts were prepared by an impregnation method. Sn_1.5_PW (0.25, 0.50, and 0.75 g) and the framework of Cu-BTC (0.50 g) at certain weight ratios were mixed in water. The obtained mixture was treated by ultrasonication for 10 min and was stirred continuously for 8 h at room temperature, followed by centrifugation and washing with distilled water three times. The resulting material was dried overnight in an oven (120°C). The synthesized Sn_1.5_PW/Cu-BTC-x hybrids with different Sn_1.5_PW to Cu-BTC weight ratios of 0.5, 1, and 1.5 were identified as Sn_1.5_PW/Cu-BTC-0.5, Sn_1.5_PW/Cu-BTC-1, and Sn_1.5_PW/Cu-BTC-1.5, respectively.

### Instrumentation

Fourier-transformed infrared spectroscopy (FTIR) spectra of the synthesized catalysts were obtained for powdered samples on KBr pellets using a PerkinElmer Spectrum 100 in the range of 400–4,000 cm^−1^. Wide-angle X-ray diffraction (XRD) patterns were recorded on a D8 ADVANCE (Germany) using CuKI (1.5406 Å) radiation to get insight into the composition of the catalysts. The morphology of the catalysts was obtained on a scanning electron microscope (SEM) at 2.0 kV (Hitachi S4800) and a transmission electron microscope (TEM) at 200 kV (FEI Tecnai G2 20). The BET surface area and pore size were determined based on nitrogen adsorption-desorption isotherms with a Quantachrome instrument (Quantachrome Instruments, Boynton Beach, USA). Thermogravimetric (TG) analysis was carried out in a NETZSCH/STA 409 PC Luxx simultaneous thermal analyzer; the samples were heated up from room temperature to 600°C at a heating rate of 5°C/min. The acidic properties of the Sn_1.5_PW/Cu-BTC-1 hybrid catalysts were characterized by temperature-programmed desorption (NH_3_-TPD) (Micromeritics AutoChem II 2920).

### Catalytic Evaluation

Using a typical approach, the esterification of OA and methanol was performed in a 50-ml stainless-steel high-pressure autoclave reactor, and an appropriate amount of catalyst was charged into the autoclave reactor. Then, the reactor was preheated in an oil bath with a magnetic stirrer at an appropriate temperature for a specific time. After completion of the reaction period, all catalysts were recovered by centrifugation at 8,000 rpm for 5–7 min and washed by anhydrous methanol. In order to calculate the OA conversion, the reactants were purified in a rotary evaporator to remove water and residual methanol. The conversion of methyl oleate was estimated by measuring the acid value of feedstock and product, and the acid value was determined according to the method described in the ISO 660-2009 standard (Animal and vegetable fats and oils—determination of acid value and acidity).

## Results and Discussion

### Catalyst Characterization

**Figure 1:**
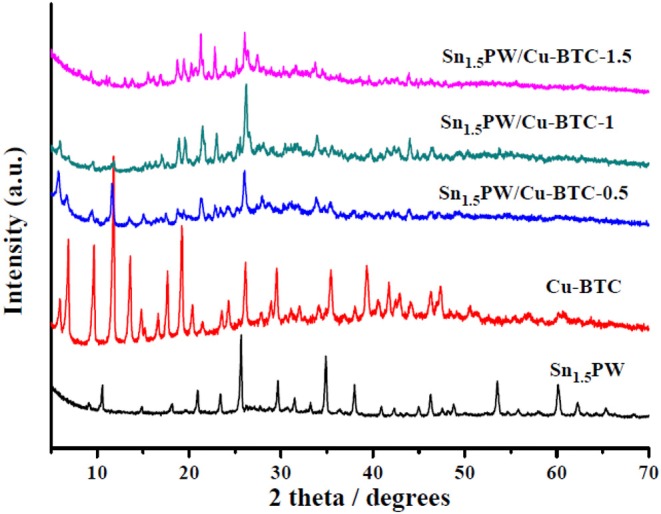
Wide-angle XRD patterns of the Sn_1.5_PW, Cu-BTC, and Sn_1.5_PW/Cu-BTC-x hybrids.

The Sn_1.5_PW, Cu-BTC, and Sn_1.5_PW/Cu-BTC-x hybrids were firstly characterized by wide-angle XRD (Figure 1). For the Sn_1.5_PW sample, the peaks at 10.5°, 14.8°, 18.2°, 20.8°, 23.3°, 25.7°, 29.7°, 35.4°, and 38.0° can be related to the Keggin unit of HPW (Pasha et al., 2019), indicating the intact Keggin ion in the Sn-exchanged HPW catalysts. According to the literature (Yang et al., 2015), the wide-angle XRD spectrum of the synthesized Cu-BTC was in perfect agreement with the spectrum of simulated Cu-BTC. After supporting Sn (II)-substituted Keggin HPW, all Sn_1.5_PW/Cu-BTC-x hybrids showed a decrease in the peak intensities of the Cu-BTC characteristic phase, and the XRD spectrum of Sn_1.5_PW could not be distinguished from the XRD spectra of Sn_1.5_PW/Cu-BTC hybrids, suggesting that the Sn_1.5_PW salts were relatively uniformly distributed on the surface of Cu-BTC cages. Interestingly, the peak intensities of Sn_1.5_PW/Cu-BTC-1 were much stronger than those of Sn_1.5_PW/Cu-BTC-0.5 and Sn_1.5_PW/Cu-BTC-1.5, which is probably due to the existence of interaction between the uniformly dispersed Sn_1.5_PW molecules and Cu-BTC matrix. These results showed that Sn_1.5_PW molecules were loaded on the surface of Cu-BTC nanocages through strong interaction.

**Figure 2:**
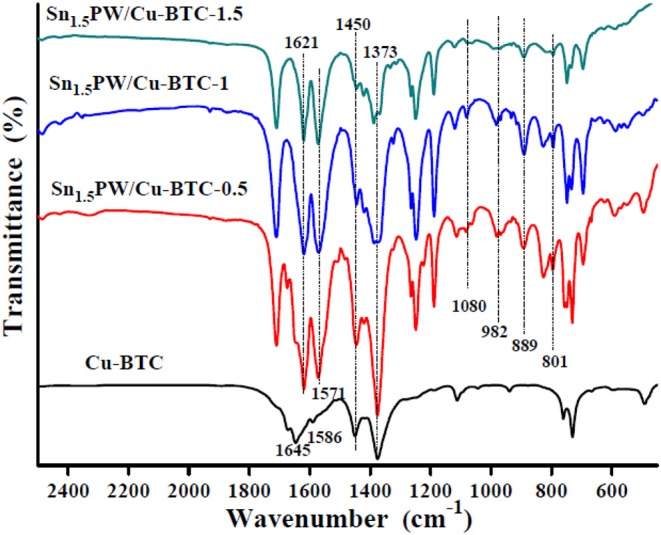
FTIR spectra of the Cu-BTC and Sn_1.5_PW/Cu-BTC-x hybrids.

The FTIR spectra of the HPW and Sn_1.5_PW samples were given in Figure S1. The FTIR spectra of HPW and Sn (II)-substituted HPW salts presented four characteristic peaks at 1,080, 982, 889, and 801 cm^−1^, which was correlated with the Keggin unit of HPW, in agreement with the literature (Zhang et al., 2016). Moreover, as can be seen in Figure 2, the FTIR spectra of all Sn_1.5_PW/Cu-BTC-x hybrids showed the peaks corresponding to Cu-BTC, the coordinated acac ligand showed peaks at 1,450 and 1,373 cm^−1^, and some characteristic peaks at 1,645 and 1,586 cm^−1^ were shifted to 1,621 and 1,571 cm^−1^, respectively, indicating strong interaction between Sn_1.5_PW and Cu-BTC nanoparticles. Surprisingly, the various Sn_1.5_PW concentrations for Sn_1.5_PW/Cu-BTC showed four peaks at 1,080, 982, 889, and 801 cm^−1^, respectively, further indicating that Sn_1.5_PW molecules were embedded around the surface of the Cu-BTC matrix. This also probably confirmed its stability and that it would suffer less leaching during the esterification reaction.

**Figure 3:**
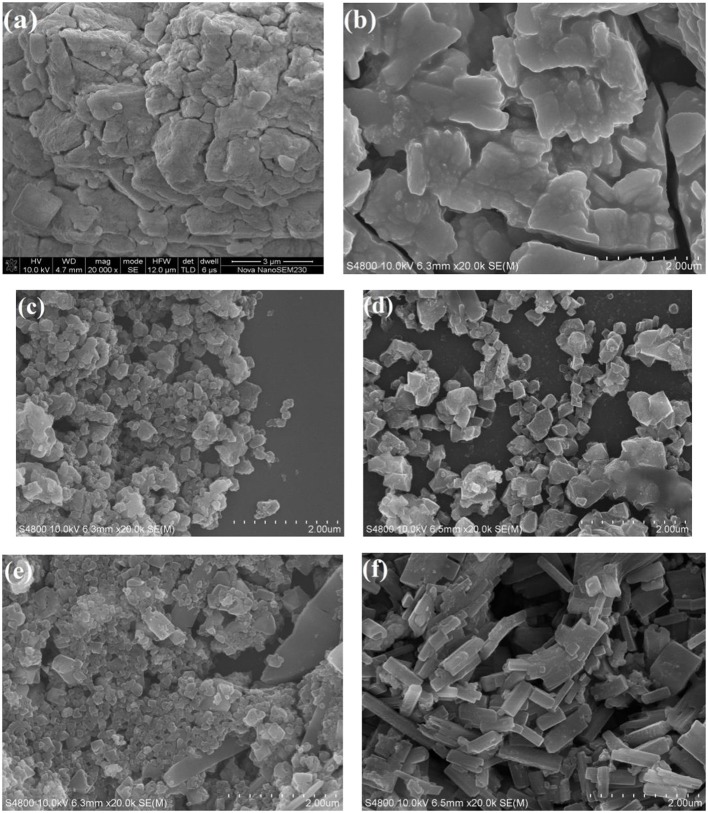
SEM images of **(a)** pure HPW, **(b)** Sn_1.5_PW, **(c)** Cu-BTC, **(d)** Sn_1.5_PW/Cu-BTC-0.5, **(e)** Sn_1.5_PW/Cu-BTC-1, and **(f)** Sn_1.5_PW/Cu-BTC-1.5.

Figure 3 shows the SEM images of the pure HPW, Sn_1.5_PW, Cu-BTC, and Sn_1.5_PW/Cu-BTC-x hybrids. The image of the pure HPW shows a large-blocked aggregate morphology. After Sn had been doped with HPW, the Sn_1.5_PW sample showed large nanoparticles of irregular shape and with a rough surface, indicating the successful exchange of protons by Sn ions, which was similar to our previously reported results (Cai et al., 2019). Moreover, Figure 3c shows a 100–200-nm size for the synthesized Cu-BTC nanoparticles, which are irregular octahedral crystals with low crystallinity and a similar morphology as observed by Na et al. (2012). When Sn_1.5_PW was supported on Cu-BTC nanoparticles, the octahedral morphologies were markedly improved, suggesting that the addition of Sn_1.5_PW can be a modulator. Of note, the images (Figures 3d–f) showed a gradual increase in the Sn_1.5_PW coating on the surface of Cu-BTC nanoparticles, and, at 0.5 g Sn_1.5_PW loading, the surface morphology of Sn_1.5_PW/Cu-BTC-1 was smooth, with no Sn1.5PW agglomeration. This observation might be due to the Cu-BTC matrix being completely coated. Meanwhile, compared to Sn_1.5_PW/Cu-BTC-0.5 and Sn_1.5_PW/Cu-BTC-1.5, the particle size of Sn_1.5_PW/Cu-BTC-1 hybrids, which was in a range of 100–250 nm, was lower; thus, the Sn_1.5_PW/Cu-BTC-1 hybrids possessed a high specific surface. Based on the above analyses, Sn_1.5_PW/Cu-BTC-1 was selected for further characterization.

**Figure 4:**
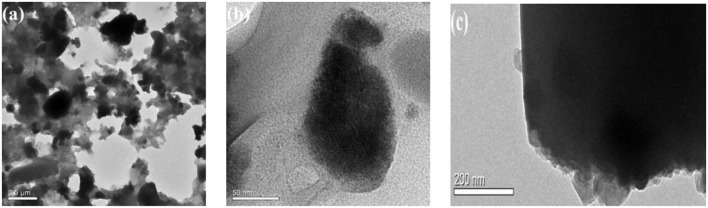
**(a–c)** Typical TEM images of the Sn_1.5_PW/Cu-BTC-1 hybrids.

To visualize Sn_1.5_PW supported on Cu-BTC, TEM images of the Sn_1.5_PW/Cu-BTC-1 were acquired; these are shown in Figure 4. From Figure 4a, it can be seen that the Sn_1.5_PW/Cu-BTC-1 presented an octahedral shape, confirming that the framework of Cu-BTC was properly retained. As highlighted in Figures 4b,c, the edges became noticeably roughened, and this reveals that many small Sn_1.5_PW particles were relatively uniformly distributed on the edges, which is consistent with the XRD and SEM results.

**Figure 5:**
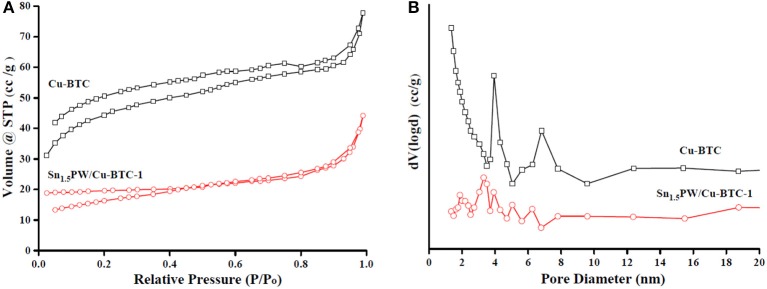
**(A)** N_2_ adsorption-desorption isotherm plots and **(B)** pore size distributions of Cu-BTC and Sn_1.5_PW/Cu-BTC-1 samples.

The N_2_ adsorption-desorption isotherms and BJH pore size distributions of Cu-BTC and Sn_1.5_PW/Cu-BTC-1 samples are shown in Figure 5. All samples show a type I isotherm, which revealed their microporous nature. Of note, the pore size distribution (Figure 5B) proved that the pores had an average diameter of 2–10 nm and a narrow size distribution. Moreover, the surface area decreased from 578.2 to 29.7 m^2^/g, and the average pore size increased from 2.38 to 7.11 nm for Cu-BTC and Sn_1.5_PW/Cu-BTC-1, respectively. The decrease in the BET surface area may be attributed to the presence of Sn_1.5_PW inside the Cu-BTC nanocages. The increase in the average pore size was probably due to a collapsed microporous structure, which was similar to previously reported results (Jeona et al., 2019). Meanwhile, the open cavities and relatively high specific surface area of Sn_1.5_PW/Cu-BTC-1 nanocomposites were retained, which made for the free diffusion of the reactants or products, consistent with SEM results.

**Figure 6:**
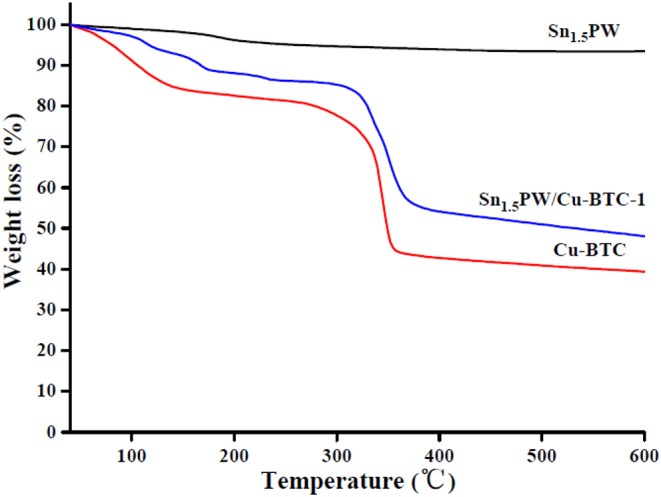
TG curves of Sn_1.5_PW, Cu-BTC, and Sn_1.5_PW/Cu-BTC-1 samples.

The thermal stabilities of the Sn_1.5_PW, Cu-BTC, and Sn_1.5_PW/Cu-BTC-1 samples were established with TG analysis (Figure 6). The Sn_1.5_PW sample showed no significant decomposition, and only 6% mass loss was observed up to 600°C. For the Cu-BTC and Sn_1.5_PW/Cu-BTC-1 samples, the TG curve exhibited two stages of mass-loss, namely 40–250 and 250–400°C; these intervals can be associated with the release of physically adsorbed water on the surface of sample and bonded water from the crystal hydrates (Azmoon et al., 2019) and the decomposition of the Cu-BTC frameworks (Xie and Wan, 2018), respectively. Above 400°C, almost no obvious mass loss was observed in the TG curve. The results indicated that the prepared catalysts had better stability and could be employed as heterogeneous catalysts for the esterification reaction.

**Figure 7:**
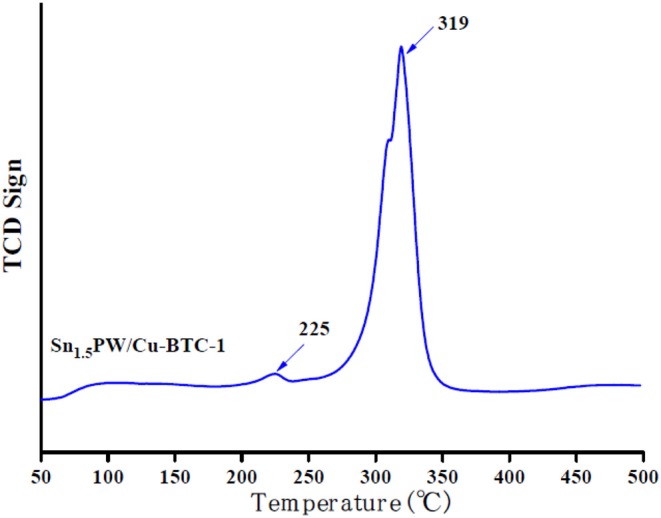
NH_3_-TPD patterns of Sn_1.5_PW/Cu-BTC-1 nanocomposite.

Figure 7 displays the NH_3_-TPD profiles of Sn_1.5_PW/Cu-BTC-1 nanocomposites. The minimum and maximum desorption temperatures of NH_3_ are 225 and 319°C. The acidity present is attributed to the surface acidity of Sn_1.5_PW. Based on these results, the nanocomposites possessed 24.6 mmol/g of total acidity. The results of NH_3_-TPD also show that the catalyst performance can be correlated with low and medium acidity strength. Therefore, the Sn_1.5_PW/Cu-BTC-1 nanocomposites exhibit higher catalytic activity in the OA esterification.

### Catalytic Performance of Different Catalysts

**Figure 8:**
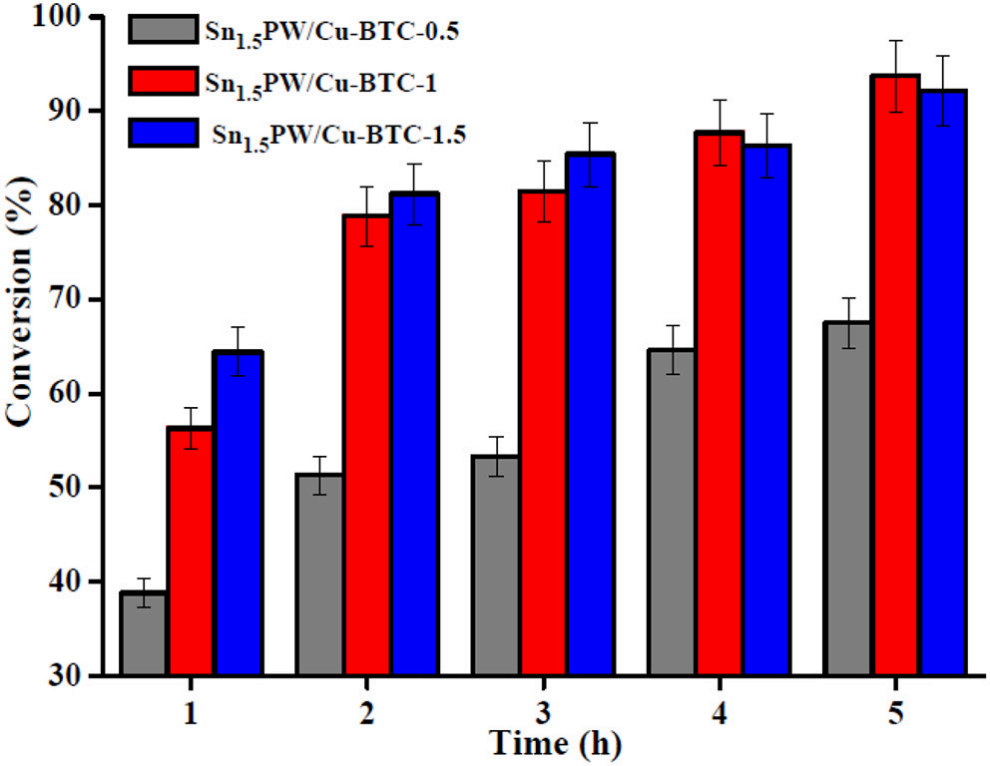
Effects of various nanocatalysts for esterification of OA.

The influence of the molar ratio of Sn_1.5_PW/Cu-BTC on the esterification of OA was studied at 160°C by using 0.2 g catalyst and a 1:20 OA to methanol molar ratio within 4 h of reaction time. The results in Figure 8 reveal that the OA conversion was enhanced with the increase of the Sn_1.5_PW/Cu-BTC ratio up to 1. The highest OA conversion was obtained by utilizing Sn_1.5_PW/Cu-BTC-1 and Sn_1.5_PW/Cu-BTC-1.5 at 5 h. Most probably, this increase in the catalytic activity can be attributed to its relatively large specific surface area and appropriate particle size. In order to save raw material and avoid Sn_1.5_PW agglomeration on the support surface, we used this Sn_1.5_PW/Cu-BTC-1 as the catalyst for further research.

### Effect of Esterification Conditions

**Figure 9:**
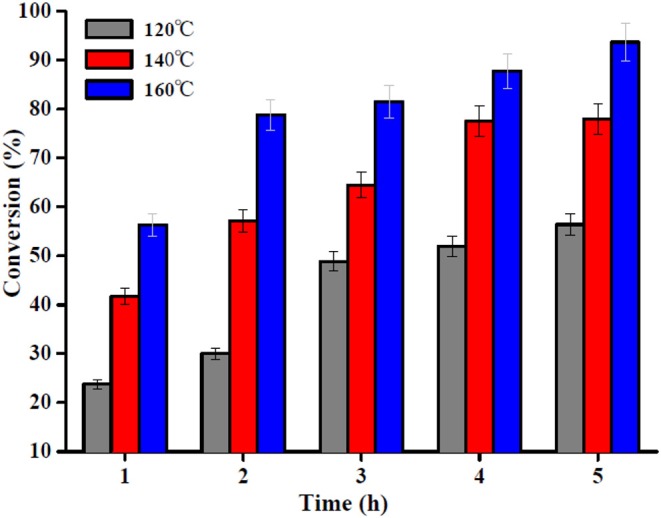
Effect of reaction time and temperature. Reaction conditions: OA/methanol molar ratio 1:20, catalyst amount 0.2 g.

Biodiesel was produced by esterification, which is a reversible reaction that converts the OA into methyl oleate (biodiesel) and water in the presence of a nanocatalyst such as the Sn_1.5_PW/Cu-BTC-1. The reaction temperature is one of the key parameters of the esterification reaction. Thus, the effect of temperature in the range 120–160°C on the esterification reaction of OA with methanol with Sn_1.5_PW/Cu-BTC-1 nanocatalyst was examined, and the results are presented in Figure 9. The results indicated that the OA conversion was improved with an increase in the temperature, which indicates that high temperature would improve the Sn_1.5_PW/Cu-BTC-1 catalytic activity due to the endothermic nature of the esterification reaction. When the temperature rose to 160°C, the OA conversion increased to 87.7% at 4 h. Moreover, the OA conversion was increased as the reaction time was increased from 1 to 4 h, but no significant increase in OA conversion was observed beyond 4 h until 5 h. Thus, the selected temperature and time for further studies were 160°C and 4 h, respectively.

**Figure 10:**
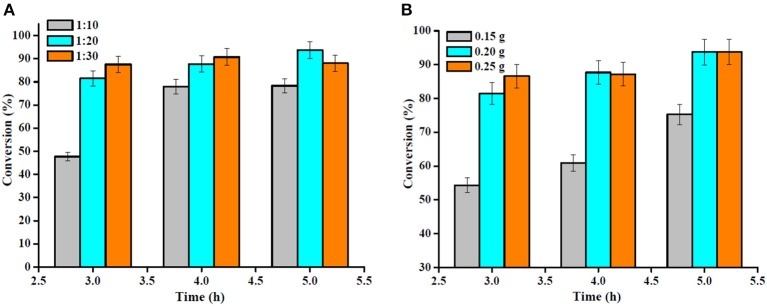
**(A)** Effect of the molar ratio of OA to methanol (reaction conditions: temperature 160°C, catalyst amount 0.2 g) and **(B)** catalyst amount (reaction conditions: temperature 160°C, OA/methanol molar ratio 1:20).

The OA to methanol molar ratio is one of the most important factors affecting the OA conversion and the cost of biodiesel production. Therefore, the effect of the OA to methanol molar ratio on OA esterification to biodiesel is shown in Figure 10A. Since esterification is a reversible reaction, high OA conversion could be achieved by using excess methanol in the reaction. It is evident that with the increase in the molar ratio of OA to methanol from 1:10 to 1:20, the OA conversion increased somewhat; however, no important change occurred as the molar ratio increased up to 1:30, and a large amount of methanol probably affected the OA conversion adversely, as the reactant was diluted and reduced the OA concentration (Nandiwale et al., 2013). Hence, the OA to methanol molar ratio was restricted to 1:20.

The study was further extended to the investigation of the effect of catalyst amount on the OA esterification reaction (Figure 10B). Under similar operating conditions, the OA conversion reached after 4 h of reaction was 60.9 and 87.7% using a Sn_1.5_PW/Cu-BTC-1 nanocatalyst amount of 0.15 and 0.20 g, respectively. This is attributed to the acceleration of the reaction rate by there being a larger number of active sites in the reaction mixture. However, when a catalyst amount of 0.25 g was used, an OA conversion of 87.2% was reached, remaining practically constant in comparison with a catalyst amount of 0.20 g. Thus, it is suggested that 0.2 g of Sn_1.5_PW/Cu-BTC-1 was the optimum amount, and this was used in the subsequent reactions.

### Kinetic Studies of Biodiesel Production Using Sn_1.5_PW/Cu-BTC-1 Nanocatalyst

Kinetic study for the OA esterification process was conducted under optimal conditions for Sn_1.5_PW/Cu-BTC-1 at three different temperatures (120, 140, and 160°C). Because an excess amount of methanol was used, the reverse reaction can be ignored, and the esterification reaction can also be assumed to follow the pseudo-first-order kinetic model (Kaur and Ali, 2015; Shalini and Chandra, 2018). Thus, the reaction rate constant is fitted in Equation (1), and the activation energy (*E*_*a*_) required for the esterification process is calculated according to the Arrhenius, Equation (2).

(1) $$\begin{array}{l} k=-\text{In}\frac{1-\eta }{t} \end{array}$$

(2) $$\begin{array}{l} \text{In}k=\text{In}A-\frac{E_{a}}{RT} \end{array}$$

where *k* is the reaction rate constant; η is the conversion of OA at time *t*; A is the Arrhenius constant or frequency factor; *R* is the universal gas constant; *T* is a reaction temperature.

**Figure 11:**
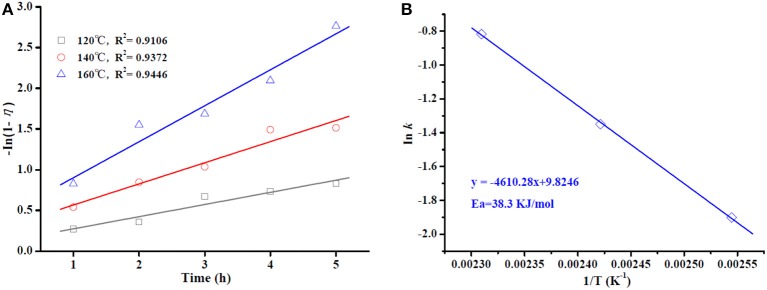
Plot of -ln (1-η) vs. reaction time at different temperatures **(A)**. Arrhenius plot of ln *k* vs. 1/T **(B)**.

A graph of ln (1-η) vs. time is given in Figure 11A. The plots display decent linearity with high regression coefficients (R^2^ = 0.9106, 0.9372, and 0.9446 for 120, 140, and 160°C, respectively), indicating that the model is appropriate in terms of pseudo-first-order kinetics. The plot of ln *k* vs. 1/T in Figure 11B is found to be linear, with a high regression coefficient (R^2^ = 0.9994). The value of *E*_*a*_ was determined to be 38.3 kJ/mol, which is much lower than the values determined in the works of Lieu et al. (2016) and Mazubert et al. (2014) for similar systems. More importantly, a value of *E*_*a*_ > 15 kJ/mol further supports that the OA esterification process in this work is controlled chemically (Patel and Brahmkhatri, 2013).

### Reusability of Catalyst

**Figure 12:**
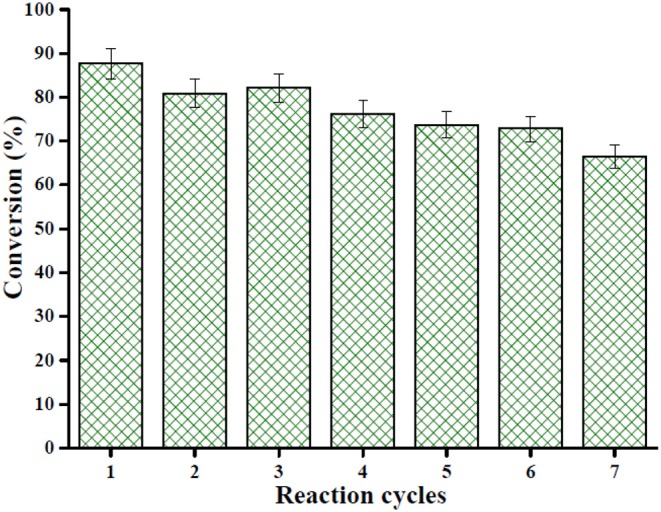
Reusability of the nanocatalyst Sn_1.5_PW/Cu-BTC-1 for seven cycles under optimum esterification conditions: temperature 160°C, catalyst amount 0.2 g, reaction time 4 h, and OA-methanol molar ratio 1:20.

**Figure 13:**
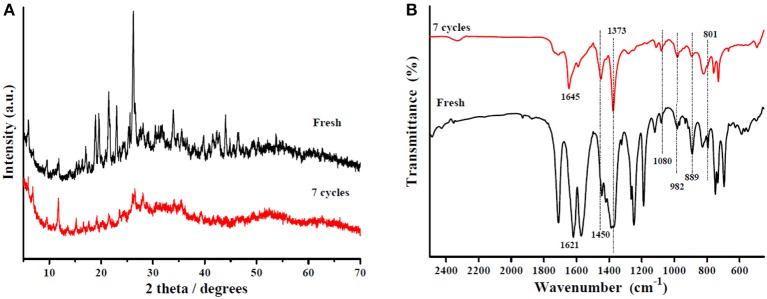
**(A)** XRD patterns of the Sn_1.5_PW/Cu-BTC-1 nanocatalyst and the nanocatalyst after seven cycles. **(B)** FT-IR spectra of the Sn_1.5_PW/Cu-BTC-1 nanocatalyst and the nanocatalyst after seven cycles.

Reusability is considered the most important characteristic of heterogeneous solid acid catalysts. In this work, the Sn_1.5_PW/Cu-BTC-1 nanocatalyst was separated by centrifugation after the completion of the reaction, washed with anhydrous methanol, and then used directly after each cycle. Under the same optimum conditions, the esterification of OA was performed seven times, and Figure 12 displays the results obtained. It was detected that the activity of the catalyst can still keep the conversion above 80% after three-time reuse. Nevertheless, it was found that, after seven times, the conversion was more than 60%. Furthermore, XRD and FT-IR tests were performed to determine the stability of the synthesized nanocatalyst; the results are presented in Figures 13A,B. According to Figure 13A, the XRD pattern of the recycled catalyst was almost consistent with that of the fresh one, and only the peak intensity decreased. As exhibited in Figure 13B, the featured peaks of the Keggin structure and the characteristic peaks of Cu-BTC were found in the FT-IR spectrum of the recycled catalyst, indicating that the Sn_1.5_PW/Cu-BTC-1 nanocatalyst had a durable structure. Based on the above discussion, the catalytic activity loss might be due to the composites in the reaction mixture losing few Sn_1.5_PW active sites. Thus, in comparison with earlier reported solid acid catalysts, the synthesized Sn_1.5_PW/Cu-BTC-1 nanocatalyst showed better stability in biodiesel synthesis.

## Conclusion

In summary, an efficient solid acid nanocatalyst, Sn_1.5_PW/Cu-BTC-1, was prepared through the immobilization of Sn_1.5_PW salts on Cu-BTC. The prepared nanocomposites were then implemented for the production of biodiesel from OA and methanol. A high conversion of 87.7% was obtained under the optimized conditions of 160°C for 4 h with a molar ratio of OA to methanol of 1:20, and the addition of 0.2 g of catalyst. In addition, this nanocomposite catalyst showed good stability, and even after seven cycles of reuse, a considerable OA conversion could still be achieved. Moreover, a pseudo-first-order kinetic model was found to represent the data more appropriately, with the *E*_*a*_ of the reaction being 38.3 kJ/mol. The remarkable point in this study was the use of a facile, simple, and cheap method for the synthesis of Sn_1.5_PW/Cu-BTC nanocomposites in the large-scale production of biodiesel.

## Data Availability Statement

The raw data supporting the conclusions of this article will be made available by the authors, without undue reservation, to any qualified researcher.

## Author Contributions

QZ was in charge of designing the experiments and writing the manuscript. DLi and DLe performed experiments. XL, JW, YZ, and PM were in charge of revising the manuscript .

### Conflict of Interest

The authors declare that the research was conducted in the absence of any commercial or financial relationships that could be construed as a potential conflict of interest.
