# Corrigendum: Green and Facile Synthesis of Metal-Organic Framework Cu-BTC-Supported Sn (II)-Substituted Keggin Heteropoly Composites as an Esterification Nanocatalyst for Biodiesel Production

**DOI:** 10.3389/fchem.2020.00292

**Published:** 2020-04-15

**Authors:** Qiuyun Zhang, Dan Ling, Dandan Lei, Jialu Wang, Xiaofang Liu, Yutao Zhang, Peihua Ma

**Affiliations:** ^1^School of Chemistry and Chemical Engineering, Anshun University, Anshun, China; ^2^Engineering Technology Center of Control and Remediation of Soil Contamination of Provincial Science & Technology Bureau, Anshun University, Anshun, China; ^3^School of Resource and Environmental Engineering, Anshun University, Anshun, China; ^4^Food and Pharmaceutical Engineering Institute, Guiyang University, Guiyang, China; ^5^School of Chemistry and Chemical Engineering, Guizhou University, Guiyang, China

**Keywords:** heteropolys, Cu-BTC, nanocomposites, esterification, biodiesel

There is an error in the Funding statement. The correct Funding Statement appears below.

## Funding

“This work was financially supported by the Guizhou Science and Technology Foundation ([2020]1Y054), the Technical Talent Support Program of Guizhou Education Department (KY [2018]069), the Academician Workstation of Guizhou Science and Technology Plan (S&T Cooperation Platform Talents [2016]5602), the Guizhou Science and Technology Cooperation Project (LH[2017]7059), the Key Support Discipline in Agricultural Resources and Environment of Anshun University, and the Creative Research Groups Support Program of Guizhou Education Department (KY [2017]049).”

The authors apologize for this error and state that this does not change the scientific conclusions of the article in any way. The original article has been updated.

