# Supplementary material for: Green and Facile Synthesis of Metal-Organic Framework Cu-BTC-Supported Sn (II)-Substituted Keggin Heteropoly Composites as an Esterification Nanocatalyst for Biodiesel Production
Source: Front Chem. 2020 Mar 18;8:129. doi: 10.3389/fchem.2020.00129 (PMC7094214; doi:10.3389/fchem.2020.00129)
Supplement: Supplementary file 1 [file Image_1.pdf]

Electronic Supplementary Information

Green and facile synthesis of metal-organic framework Cu-BTC-supported Sn (II)-substituted Keggin heteropoly composites as esterification nanocatalyst for biodiesel production

Qiuyun Zhang [a,b]*, Dan Ling[a], Dandan Lei[a], Jialu Wang[c] *, Xiaofang Liu[d], Yutao Zhang [b,c], and Peihua Ma [e] *

[*a*] School of Chemistry and Chemical Engineering, Anshun University, China
[*b*] Engineering Technology Center of Control and Remediation of Soil Contamination of Provincial Science & Technology Bureau, Anshun University, China
[c] School of Resource and Environmental Engineering, Anshun University, China
[d] Food and Pharmaceutical Engineering Institute, Guiyang University, China
[e] School of Chemistry and Chemical Engineering, Guizhou University, China

* Correspondence: qyzhang.asu@gmail.com (Qiuyun Zhang); lu226@163.com (Jialu Wang); phma@gzu.edu.cn (Peihua Ma).

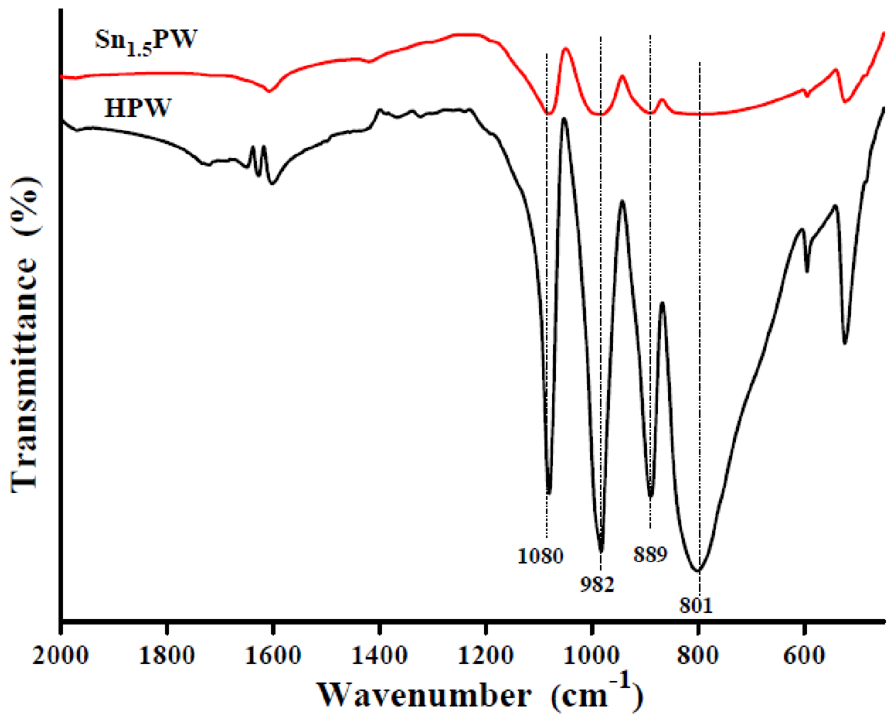

Figure S1 FTIR spectra of the pure HPW and $Sn_{1.5}PW$ sample
